# Clinical manifestations and outcome of viral acute lower respiratory infection in hospitalised children in Myanmar

**DOI:** 10.1186/s12879-022-07342-1

**Published:** 2022-04-08

**Authors:** Kazuhiro Kamata, Khin Nyo Thein, Lasham Di Ja, Nay Chi Win, Su Mon Kyaw Win, Yuko Suzuki, Ai Ito, Hidekazu Osada, Irina Chon, Wint Wint Phyu, Yuta Aizawa, Tatsuki Ikuse, Tomomi Ota, Yadanar Kyaw, Htay Htay Tin, Yugo Shobugawa, Hisami Watanabe, Reiko Saito, Akihiko Saitoh

**Affiliations:** 1 Infectious Diseases Research Center of Niigata University in Myanmar, Yangon, Myanmar; 2 Department of Pediatrics, Niigata University Graduate School of Medical and Dental Sciences, 1-757 Asahimachi-dori, Chuo-ku, Niigata, Niigata 951-8510 Japan; 3 Yankin Children Hospital, Yangon, Myanmar; 4 Division of International Health, Graduate School of Medical and Dental Science, Niigata University, Niigata, Japan; 5 Respiratory Medicine Department, Thingangyun Sanpya General Hospital, Yangon, Myanmar; 6 Department of Medical Services, National Health Laboratory, Ministry of Health and Sports, Yangon, Myanmar

**Keywords:** Acute lower respiratory infection, Virus, Children, Respiratory syncytial virus, Influenza, Myanmar

## Abstract

**Background:**

Acute lower respiratory infection (ALRI) remains the leading cause of death in children worldwide, and viruses have been the major cause of ALRI. In Myanmar, ALRI is associated with high morbidity and mortality in children, and detailed information on ALRI is currently lacking.

**Methods:**

This prospective study investigated the viral aetiologies, clinical manifestations, and outcomes of ALRI in hospitalised children aged 1 month to 12 years at the Yankin Children Hospital, Yangon, Myanmar from May 2017 to April 2019. The sample size was set to 300 patients for each year. Two nasopharyngeal swabs were obtained for the patients with suspected viral ALRI; one for rapid tests for influenza and respiratory syncytial virus (RSV), and the other for real-time PCR for the 16 ALRI-causing viruses. Pneumococcal colonization rates were also investigated using real-time PCR. Clinical information was extracted from the medical records, and enrolled patients were categorised by age and severity for comparison.

**Results:**

Among the 5463 patients admitted with a diagnosis of ALRI, 570 (10.4%) were enrolled in this study. The median age of the patients was 8 months (interquartile range, 4–15 months). The most common symptoms were cough (93%) and difficulty in breathing (73%), while the most common signs of ALRI were tachypnoea (78%) and chest indrawing (67%). A total of 16 viruses were detected in 502 of 570 patients’ samples (88%), with RSV B (36%) and rhinovirus (28%) being the most commonly detected. Multiple viruses were detected in 221 of 570 samples (37%) collected from 570 patients. Severe ALRI was diagnosed in 107 of 570 patients (19%), and RSV B and human rhinovirus were commonly detected. The mortality rate was 5%; influenza virus A (29%) and RSV B (21%) were commonly detected, and stunting and lack of immunization were frequently observed in such cases. Additionally, 45% (259/570) of the patients had pneumococcal colonization.

**Conclusions:**

Viral ALRI in hospitalised children with a median of 8 months has significant morbidity and mortality rates in Myanmar. RSV and rhinovirus were the most commonly detected from nasopharyngeal swabs, while influenza virus and RSV were the most frequently associated with fatal cases.

**Supplementary Information:**

The online version contains supplementary material available at 10.1186/s12879-022-07342-1.

## Background

Acute lower respiratory infection (ALRI), which comprises bronchitis, bronchiolitis, and pneumonia, remains the leading cause of death worldwide, accounting for 10% of deaths in children younger than 5 years [1–3]. Remarkable progress in ALRI prevention and treatment has been achieved in the last few decades in developing countries. Key factors include the improvement of nutrition and sanitation, distribution and use of antibiotics and antivirals for influenza, and global promotion of the use of *Haemophilus influenzae* type b (Hib) vaccine and pneumococcal conjugate vaccines (PCV) in infants and children [4–9]. Globally, between 2000 and 2015, the incidence of pneumonia among children younger than 5 years decreased by 30% (178 million in 2000 to 138 million in 2015), and the number of deaths in young children was almost halved (1.7 million in 2000 to 0.9 million in 2015) [4, 5, 10]. However, ALRI has remained the infectious disease with the highest mortality in children, with more than 95% of deaths occurring in low- and middle-income countries [11–13]. Thus, continuous efforts and future strategies are needed to overcome the high mortality caused by ALRI in children.

ALRI in children is caused by various pathogens, including bacteria (e.g. *Streptococcus pneumoniae*, *Haemophilus influenzae*) and viruses (e.g. respiratory syncytial virus [RSV], influenza virus, etc.) [5]. However, recent studies in the United States have demonstrated that most pneumonia cases in children were caused by a small set of viral pathogens, not bacteria [14–16]. This shift has also been observed in some developing countries in Asia and Africa, including Bangladesh, India, Kenya, and Zambia [14, 16, 17], as viral pathogens, including RSV, human rhinovirus (hRV), human metapneumovirus (hMPV), human bocavirus (hBoV), parainfluenza viruses (PIV), and influenza virus, have been frequently identified in children with ALRI [17–19]. It is assumed that the improvement and widespread availability of molecular diagnostics and the extensive immunisation programs for Hib and PCV have accelerated this trend, increasing the relative importance of viral aetiologies in children with ALRI [20]. Notably, viral diagnosis has become extremely important during the global pandemic caused by the coronavirus disease in 2020 [21].

Myanmar is a lower-middle-income country in Asia that has been developing and expanding significantly after the political reform and democratisation in 2011. The Hib vaccine and 10-valent PCV were introduced in 2012 and 2016, respectively [22]. Both of the vaccines are recommended at ages 2, 4, and 6 months as routine immunization without a fourth (booster) dose [23]. The immunisation coverage rates in 2017 were estimated to be approximately 90% for the third dose of both vaccines [24], although actual vaccination rates might be much lower, especially in rural areas. Nevertheless, the serious issue of high childhood mortality rate is still present in Myanmar as the under-five mortality rate in 2016 was 51 per 1,000 live births, the majority of which was attributable to ALRI, diarrhoea, or septicaemia [25, 26]. However, little has been reported on ALRI in children in Myanmar. Based on the data collected from the refugees living on the Thailand-Myanmar border [27], the influenza virus has a strong link to neighbouring countries [28], and RSV and influenza virus were frequently detected in children less than 5 years and equal or greater than 5 years, respectively [27].

To better understand the viral aetiologies, clinical manifestations, and clinical outcomes of ALRI in children in Myanmar, we performed a prospective study in one of the largest children's hospitals in Yangon, Myanmar. The results obtained in this study will lead to better strategies in preventing and/or treating viral ALRI and decreasing its associated morbidity and mortality in Myanmar.

## Methods

### Study design and study population

This prospective study investigated the clinical characteristics, clinical outcomes, and viral aetiologies in children in Myanmar who were admitted to the Yankin Children Hospital (YKCH) in Yangon, Myanmar for ALRI from April 2017 to March 2019. YKCH, affiliated with the University of Yangon, Medicine II, is one of the largest children's hospitals in Yangon with 550 beds. The hospital plays a central role in the primary to tertiary care of children in the Yangon metropolitan area, which has a childhood population of more than 1.7 million [29]. We enrolled patients aged 1 month to 12 years who were admitted to the general wards or the intensive care unit at YKCH under the diagnosis of ALRI. The maximum age at admission at YKCH was 12 years. We asked each paediatrician to recruit certain numbers of patients each month to investigate the viral cause of ALRI throughout the study period.

Based on the World Health Organization (WHO) definition [30, 31], ALRI was defined as (1) history or measured fever of ≥ 38 °C, (2) cough, (3) onset within the last 10 days, and (4) hospitalisation requirement. Particularly for patients aged < 5 years, ALRI was defined as (1) fast breathing: less than 2 months: ≥ 60 bpm, 2 months to less than 12 months: ≥ 50 bpm, 12 months to less than 5 years: ≥ 40 bpm, or (2) difficulty of breathing with chest indrawing and cough.

With regards to the patient characteristics, stunting was categorised using the height-for-age z-score: normal, ≥ − 2; moderate, ≥ − 3 to < − 2; severe, < − 3 [26]. Tachycardia was defined as a heart rate ≥ 160 bpm in children aged < 1 year and ≥ 120 bpm in children aged ≥ 1 year [19]. Hypoxemia was defined as peripheral oxygen saturation (SpO_2_) ≤ 90% by pulse oximetry [19, 31]. Tachypnea was defined as a respiratory rate of ≥ 60 bpm in children aged < 2 months, ≥ 50 bpm in children aged 2–11 months, and ≥ 40 bpm in children aged ≥ 1 year [19, 30, 31]. Fever was defined as a body temperature ≥ 38.0 °C [19].

The laboratory examinations, including blood tests, bacterial culture, and chest radiographs, were ordered according to the physicians’ discretion. Radiological findings were evaluated by senior paediatricians using an endpoint of consolidation, over-inflation, and whether or not the findings were unilateral or bilateral. Admission criteria were based on each physician’s decision. However, the majority of the reasons for admission were (1) requirement of supplemental oxygen due to hypoxia, (2) requirement of hydration due to intolerable oral feeding, and/or (3) poor general condition.

Exclusion criteria for this study included those who (1) were recently hospitalised with acute respiratory illness within 7 days, (2) had already been enrolled in this study < 28 days, (3) diagnosed with bacterial ALRI by physical examination and microbiological tests (blood, nasopharyngeal and oropharyngeal swab, or sputum cultures); (4) had an alternative diagnosis of other respiratory disorders; (5) had a tracheostomy tube; or (6) had baseline diseases causing immunocompromised or altered respiratory status, such as chromosomal abnormalities, cancer, known respiratory diseases (bronchiectasis, cystic fibrosis, etc.), cerebral palsy, and human immunodeficiency virus infection.

All patients were conclusively diagnosed with ALRI by senior paediatricians. They also interviewed enrolled children and their caregivers using standardised questionnaires to collect information regarding the baseline characteristics and medical history. Data from medical records were abstracted systematically after discharge.

### Ethics approval and consent to participate

All methods used in the current study were performed in accordance with the relevant guidelines and regulations. Written informed consent was obtained from all the study participants, including their parents and guardians, before participation in the study. This study was approved by the ethics committees of the Department of Medical Research, Ministry of Health and Sports, Myanmar (016616) and Niigata University, Japan (2547).

### Definition of severe ALRI

Severe ALRI was defined when children with ALRI had (1) central cyanosis or SpO_2_ ≤ 90%, (2) severe respiratory distress, or 3) “general danger signs,” such as inability to drink, persistent vomiting, convulsions, or unconsciousness [30, 31]. The WHO definition was designed for children aged 2 months to 5 years, but we adapted the definition to 1 month to 12 years to simplify the study criteria at the study site.

### Nasopharyngeal sampling and microbiological analyses

At the time of admission, paediatricians collected two nasopharyngeal swabs from each patient who satisfied the case definition and agreed to participate in the study. One swab was used for testing with a rapid test kit (Quick Navi-Flu + RSV, Denka Seiken Co. Ltd., Tokyo, Japan) at the medical ward at YKCH. The other swab was immediately placed in viral transport media, and stored at − 80 °C in a freezer at YKCH and subsequently transported to the laboratory of Niigata University, Japan for real-time reverse transcription PCR (RT-PCR). Both tests were performed for all the patients regardless of the rapid test results. The RT-PCR test results were used to estimate the sensitivity and specificity of the rapid tests for influenza and RSV.

After thawing, the samples were centrifuged at 1500 × *g* for 30 min at 4 °C, and the supernatants were used in the analysis. Viral RNA and DNA were extracted using a QIAamp MinElute Virus Spin Kit (QIAGEN, Valencia, CA, USA). Real-time reverse transcription PCR (RT-PCR) was performed using the One Step PrimeScript RT-PCR Kit (TaKaRa, Tokyo, Japan) with virus-specific primers and a TaqMan probe. For the RNA virus, the thermocycling settings were as follows: 42 °C for 5 min for cDNA synthesis, 95 °C for 3 min followed by 45 cycles at 95 °C for 5 s for denaturation, and 60 °C for 40 s for annealing and extension, except for human coronavirus (hCoV), which was under different conditions (46 °C for 40 s for the annealing and extension process). Regarding the DNA virus, the thermocycling settings were as follows: 95 °C for 3 min followed by 45 cycles at 95 °C for 5 s for denaturation, and 60 °C for 40 s for annealing and extension. Using this PCR assay with recorded cycle threshold (Ct) values to estimate relative RNA or DNA levels, 16 viruses were examined: RSV (RSV A and RSV B), hRV, influenza virus (influenza A and influenza B), hMPV, enteroviruses, PIV (PIV 1–3), human coronavirus (hCoV; NL63, OC43, 229E, and HKU), adenovirus, and hBoV [32–41]. The primers and probes with concentrations used for each virus in the study are summarized in Additional file 1: Table S1. If the Ct value was above 35, we confirmed the existence of target bands in the PCR products by gel electrophoresis. The presence of target bands was confirmed as positive results.

In addition to the 16 viruses, a real-time PCR assay for *Streptococcus pneumoniae* [42–44] was performed to estimate colonisation rates. Real-time PCR was performed using the specific primers designed to target the following genes: *cps*-A and Takara SYBR Premix Ex Taq II (Product code: RR820A; TaKaRa, Tokyo, Japan). The thermocycling conditions were as follows: 95 °C for 30 s, followed by 40 cycles at 95 °C for 5 s for denaturation, and 60 °C for 30 s for annealing and extension.

Bacterial culture, including blood, nasopharyngeal and oropharyngeal swab, or sputum, was obtained based on their availability when each physician suspected bacterial ALRI. Bacterial culture was repeatedly obtained when bacterial ALRI was suspected in patients who enrolled the study.

### Study outcomes

The primary outcome of this study was to clinically characterise the ALRI patients with in-hospital mortality. The secondary outcome was the identification of viral pathogens that caused ALRI.

### Sample size calculation

Based on the YKCH statistics from 2015 to 2016, the annual inpatient admission of ALRI was reported to be approximately 3000 cases. The sample size was set to 300 patients each year, and we tried to recruit the patients each month to observe the pathogens causing ALRI at different time points in 2 years.

### Statistical analyses

Paper-based survey data were entered independently and in duplicate by the study team. Data are reported as the median and interquartile range (IQR) or as the mean and standard deviation (SD) or percentages, where appropriate. Categorical variables were analysed using the χ^2^ test, and continuous variables were assessed using the Mann–Whitney *U*-test. Statistical significance was set at p < 0.05. Statistical testing was performed using the Statistical Package for the Social Sciences (ver. 24) (IBM, Armonk, NY, USA).

## Results

### Baseline characteristics of the ALRI patients (Table 1)

**Fig. 1:**
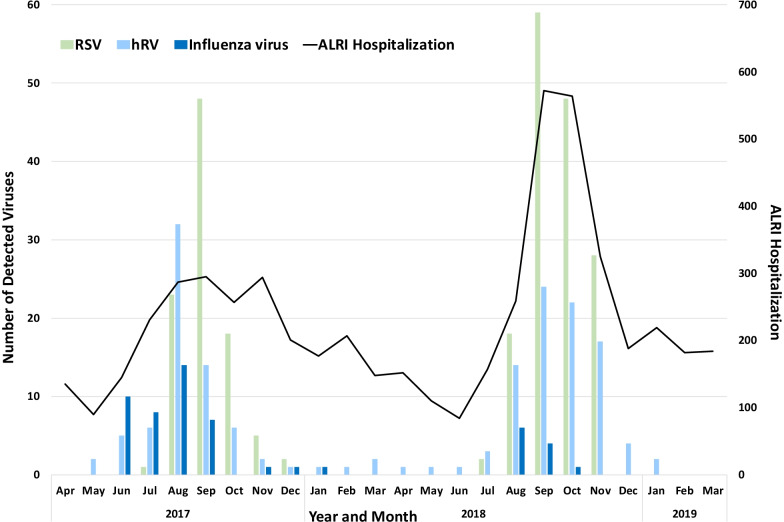
Numbers of the detected viruses in admitted patients with acute lower respiratory infection. The total number of patients hospitalised under the diagnosis of acute lower respiratory infection in each month during the study period is shown (black line). Commonly detected viruses, including respiratory syncytial virus (light green bars), human rhinovirus (light blue bars), and influenza virus (blue bars) are independently shown. *If two or more viruses were detected in one patient, each detected virus was counted and accumulated in the bars. *RSV* respiratory syncytial virus, *hRV* human rhinovirus, *ALRI* acute lower respiratory infection

In total, 5463 children diagnosed with ALRI were admitted to the YKCH between 1 April 2017 and 30 March 2019. The average number of admissions was 228 patients/month, and the number of patients admitted in 2017 and 2018 was 2,467 and 2,996, respectively. The number of ALRI cases peaked during the rainy season in both years, with the highest peak observed in September (solid line, Fig. 1). Of these, 570 (10.4%) eligible children were enrolled in the study. The clinical characteristics of the patients classified by age, severity, and detected number of viruses are summarised in Table 1 and Additional file 1: Table S2. The median age of the patients was 8 months (IQR: 4–15 months), and 56.5% were male. The patients were divided into the following 3 age groups; (1) 1–11 months, (2) 1–5 years, and (3) 6–12 years. Moderate to severe stunting (weight for age Z score < − 2) was observed in 138 (25%) patients. Among the eligible patients, 49.6% (n = 262) and 70.5% (n = 372) had received the 10-valent PCV10 and Hib vaccines (n = 528), respectively. Sick contact was observed in only 11% (65/570) of the patients. With regards to the time from the onset of disease, 70% (401/507) visited the hospital and received health care equal to or greater than 3 days after the onset of symptoms. The most common symptoms were cough (94%) and difficulty of breathing (73%).

**Table 1:** Clinical characteristics of patients with ALRI according to age and severity (n = 570)

		Total N (%)	Age category (%)			Case category (%)		
			1–11 months old	1–5 years old	6–12 years old	Non-severe cases	Severe cases	Fatal cases
Total N (%)		570	381 (67)	178 (31)	11 (2)	463 (81)	107 (19)	28 (5)
Characteristics	Median age [months] [IQR]	8.0 [4.0–15.0]	5.0 [3.0–8.0]	20.0 [15.0–31.0]	84.0 [78.5–102.0]	8.0 [4.0–16.0]	8.0 [3.0–12.0]	7.0 [3.0–21.0]
	Male	322 (56)	221 (58)	94 (53)	7 (64)	274 (59)	48 (45)	11 (39)
	Weight-for-age Z score							
	Severe (< − 3)	66 (12)	40 (10)	24 (13)	2 (18)	53 (11)	13 (12)	7 (25)
	Moderate (≥ − 3 to < − 2)	72 (13)	38 (10)	32 (18)	2 (18)	59 (13)	13 (12)	4 (14)
	Normal (≥ − 2)	432 (76)	303 (80)	122 (69)	7 (64)	351 (76)	81 (76)	17 (61)
	Underling diseases	73 (13)	43 (11)	29 (16)	1 (9)	48 (10)	25 (23)	10 (36)
	Smoker in family	243 (43)	163 (43)	75 (42)	5 (45)	183 (40)	60 (56)	15 (54)
Immunization	DPT	422 (74)	259 (68)	154 (87)	9 (82)	354 (76)	68 (64)	14 (50)
	Hib	374 (66)	235 (62)	132 (74)	7 (64)	314 (68)	60 (56)	11 (39)
	BCG	460 (81)	295 (77)	157 (88)	8 (73)	387 (84)	73 (68)	14 (50)
	MR	138 (24)	33 (9)	99 (56)	6 (55)	115 (25)	23 (22)	6 (21)
	PCV	264 (46)	165 (43)	95 (53)	4 (36)	223 (48)	41 (38)	9 (32)
	None	72 (13)	61 (16)	11 (6)	1 (9)	48 (10)	24 (22)	9 (32)
History	Sick contact	65 (11)	44 (12)	20 (11)	1 (9)	51 (11)	14 (13)	5 (18)
	Symptoms onset [Days] [IQR]	3.0 [1.5–4.0]	3.0 [2.0–4.0]	2.0 [1.0–4.0]	3.0 [2.3–4.8]	2.0 [1.0–4.0]	3.0 [2.0–4.0]	4.0 [2.0–6.0]
Symptoms	Cough	534 (94)	357 (94)	168 (94)	9 (82)	432 (93)	102 (95)	26 (93)
	Difficult breathing	415 (73)	287 (75)	121 (68)	7 (64)	324 (70)	91 (85)	28 (100)
	Rhinorrhea	247 (43)	161 (42)	82 (46)	4 (36)	205 (44)	42 (39)	16 (57)

All categorical data are presented as numbers (percentage, %). Continuous data are presented as median (interquartile range)

*ALRI* acute lower respiratory infection, *Total N* total number, *IQR* interquartile range, *DPT* a combination vaccine of diphtheria, pertussis, and tetanus, *Hib* *Haemophilus influenzae* type b vaccine, *BCG* Bacille Calmette-Guérin vaccine, *MR* Measles-rubella vaccine, *PCV* pneumococcal conjugate vaccines

### Physical examination and clinical course (Table 2 and Additional file 1: Table S3)

Among the vital signs, tachypnoea was the most common finding (78%), with a median of 60 bpm (IQR: 48–64 bpm), followed by fever (≥ 38.0 °C) (41%), and tachycardia (36%) (Table 2). On physical examination, chest indrawing, rhonchi, and coarse crackles were commonly observed in 67%, 63%, and 51% of the patients, respectively. Symptoms and signs divided by z-score did not differ significantly (P ≥ 0.05).

**Table 2:** Clinical signs, course, and outcomes of children with ALRI according to age and severity (n = 570)

		Total N (%)	Age category (%)			Case category (%)		
			1–11 months old	1–5 years old	6–12 years old	Non-severe cases	Severe cases	Fatal cases
Total N (%)		570	381 (67)	178 (31)	11 (2)	463 (81)	107 (19)	28 (5)
Vital signs	Tachycardia	208 (36)	85 (22)	116 (65)	7 (64)	147 (32)	61 (57)	18 (64)
	Heart rate [/m] [IQR]	140 [122–152]	140 [128–156]	132 [120–150]	120 [120–150]	135 [120–148]	160 [140–164]	160 [140–168]
	Tachypnoea	443 (78)	280 (73)	155 (87)	8 (73)	344 (74)	99 (93)	27 (96)
	Respiratory rate [/m] [IQR]	60 [48–64]	60 [52–64]	56 [44–64]	49 [42–55]	56 [48–62]	64 [60–70]	69 [62–78]
	Fever ≥ 38℃	232 (41)	137 (36)	90 (51)	5 (45)	172 (37)	60 (56)	19 (68)
	Body temperature [℃] [IQR]	37.8 [37.2–38.3]	37.8 [37.2–38.3]	38.0 [37.2–38.7]	38.0 [37.3–38.5]	37.8 [37.2–38.3]	38.0 [37.7–38.8]	38.7 [37.8–39.0]
	Hypoxaemia ≤ 90%	70 (12)	47 (12)	21 (12)	2 (18)	0 (0)	70 (65)	15 (54)
	Oxygen saturation [%] [IQR]	96 [94–98]	96 [94–98]	97 [95–98]	95 [93–98]	97 [95–98]	89 [85–94]	92 [80–98]
Clinical signs	Chest indrawing	383 (67)	260 (68)	116 (65)	7 (64)	290 (63)	93 (87)	24 (86)
	Coarse crackles	291 (51)	191 (50)	95 (53)	5 (45)	206 (44)	85 (79)	26 (93)
	Wheezing	212 (37)	145 (38)	63 (35)	4 (36)	160 (35)	52 (49)	17 (61)
	Rhonchi	357 (63)	256 (67)	99 (56)	2 (18)	291 (63)	66 (62)	15 (54)
	Asymmetry of lung sounds	18 (3)	8 (2)	8 (4)	2 (18)	6 (1)	12 (11)	5 (18)
	Normal lung sounds	29 (5)	11 (3)	17 (10)	1 (9)	26 (6)	3 (3)	1 (4)
	Grunting	130 (23)	99 (26)	30 (17)	1 (9)	63 (14)	67 (63)	21 (75)
	Cyanosis	65 (11)	44 (12)	21 (12)	0 (0)	0 (0)	65 (61)	20 (71)
	Capillary refill time ≥ 3 s	50 (9)	30 (8)	19 (11)	1 (9)	25 (5)	25 (23)	11 (39)
Treatment	Antibiotics	325 (57)	204 (54)	114 (64)	7 (64)	236 (51)	89 (83)	28 (100)
	2 or more than 2 drugs	107 (19)	71 (19)	33 (19)	3 (27)	64 (14)	43 (40)	17 (61)
	Anti-viral treatment	12 (2)	4 (1)	7 (4)	1 (9)	11 (2)	1 (1)	0 (0)
	ICU management	51 (9)	32 (8)	18 (10)	1 (9)	12 (3)	39 (36)	14 (50)
	O_2_ supply	328 (58)	232 (61)	89 (50)	7 (64)	231 (50)	97 (91)	27 (96)
	Mechanical ventilation	39 (7)	25 (7)	13 (7)	1 (9)	7 (2)	32 (30)	12 (43)
	CPAP	14 (2)	14 (4)	0 (0)	0 (0)	8 (2)	6 (6)	3 (11)
	Fluid infusion	176 (31)	122 (32)	48 (27)	6 (55)	115 (25)	61 (57)	24 (86)
Outcomes	In-hospital mortality	28 (5)	18 (5)	10 (6)	0 (0)	8 (2)	20 (19)	28 (100)
	Sequelae	8 (1)	5 (1)	3 (2)	0 (0)	5 (1)	3 (3)	–
	Length of hospitalization [day][IQR]	4.0 [3.0–7.0]	4.0 [3.0–6.0]	4.0 [3.0–7.0]	7.0 [4.0–8.5]	4.0 [3.0–6.0]	7.0 [4.0–10.0]	5.5 [1.0–12.0]

All categorical data arere presented as numbers (percentage, %). Continuous data are presented as median (interquartile range)

*ALRI* acute lower respiratory infection, *Total N* total number, *IQR* interquartile range, *ICU* intensive care unit, *CPAP* continuous positive airway pressure

Patients with ALRI were admitted to the ward for a median of 4 days (IQR, 3–7 days). Intensive care unit management was required in 51 patients (9%). The in-hospital mortality rate was 5% (28/570).

Although a viral infection was suspected, antibiotic therapy was initiated in more than half of the patients (325/570, 57%) based on each peadiatrician’s decision, not by a protocol or guideline for the use of antibiotics in the hospital. Among them, 107 (33%) patients received two or more antibiotics during hospitalisation (Table 2), and the simultaneous use of two or more antibiotics was documented in 91 (28%) patients. Third-generation cephalosporins (55%) and amoxicillin/clavulanic acid (34%) were commonly prescribed, followed by amoxicillin/flucloxacillin (13%), cefoperazone/sulbactam (9%), ampicillin or penicillin G (7%), piperacillin/tazobactam (6%), azithromycin (4%), carbapenems (3%), and quinolones (2%).

### Laboratory and radiologic findings (Tables 3, 4 and Additional file 1: Table S4)

A complete blood count was obtained in 63% of the patients. The median white blood cell count was 13.4 × 10^3^/μL (IQR: 9.3 × 103/μL) (Table 3). C-reactive protein was measured in 41% of the patients and had a median of 1.6 mg/dL (IQR: 0.3–4.6 mg/dL). A chest radiograph was obtained in 59% of the patients, and consolidation and over-inflation were observed in 44% and 43% of the patients, respectively. Rapid diagnostic tests for both RSV and influenza were performed in 99% of the patients, with 128 (23%) and 23 (4%) patients testing positive for RSV and influenza, respectively (Table 4).

**Table 3:** Laboratory and radiologic findings in patients ALRI according to age and severity (N = 570)

			Age category (%)			Case category (%)		
		Total N (%)	1–11 months old	1–5 years old	6–12 years old	Non-severe cases	Severe cases	Fatal cases
Total N (%)		570	381 (67)	178 (31)	11 (2)	463 (81)	107 (19)	28 (5)
CBC	Numbers of tests performed (%)	361 (63)	224 (59)	128 (72)	9 (82)	272 (59)	89 (83)	27 (96)
	WBC [× 10^3^/μL] [IQR]	13.4 [9.7–18.6]	13.6 [10.0–18.1]	12.6 [9.0–18.4]	22.0 [10.5–26.1]	12.7 [9.3–17.3]	15.9 [12.0–21.0]	20.0 [12.9–24.9]
	Neutrophil [%]	50 [37–69]	50 [36–69]	50 [37–69]	51 [37–69]	50 [37–69]	50 [36–69]	51 [38–69]
	Hemoglobin [g/dL] [IQR]	10.4 [9.6–11.2]	10.3 [9.5–11.1]	10.8 [9.8–11.5]	10.4 [9.5–11.0]	10.4 [9.7–11.2]	10.3 [9.4–11.2]	10.4 [9.2–11.5]
	Platelets [× 10^3^/µL] [IQR]	393 [285–507]	412 [308–533]	366 [255–452]	332 [277–479]	394 [293–500]	389 [249–521]	277 [182–480]
Chemistry	Numbers of tests performed (%)	231 (41)	133 (35)	90 (51)	8 (73)	171 (37)	60 (56)	19 (68)
	C-reactive protein [mg/dL][IQR]	1.6 [0.3–4.6]	1.3 [0.2–3.8]	1.9 [0.5–4.7]	9.5 [0.6–11.9]	1.5 [0.2–4.2]	1.7 [0.5–5.5]	1.8 [0.8–5.2]
Chest X-ray	Numbers of tests performed (%)	336 (59)	209 (55)	118 (66)	9 (82)	250 (54)	86 (80)	25 (89)
	Consolidation	148 (44)	86 (41)	57 (48)	5 (56)	88 (35)	60 (70)	20 (80)
	Overinflation	144 (43)	92 (44)	50 (42)	2 (22)	117 (47)	27 (31)	3 (12)
	Normal Finding	36 (11)	26 (12)	9 (8)	1 (11)	34 (14)	2 (2)	0 (0)

Categorical data are presented as number (percentage, %). Continuous data are presented as median (interquartile range)

*ALRI* acute lower respiratory infection, *Total N* total number, *IQR* interquartile range, *CBC* complete blood count, *WBC* white blood cells, *RSV* respiratory syncytial virus, *PCR* polymerase chain reaction

**Table 4:** Sensitivity and specificity of the rapid diagnostic tests for RSV and influenza virus

		PCR			Sensitivity (%)	Specificity (%)
		Positive	Negative	Total		
Rapid Test Kits for RSV	Positive	109	19	128	43	94
	Negative	142	295	437		
	Total	251	314	565		
Rapid Test Kits for Influenza virus	Positive	23	0	23	44	100
	Negative	29	513	542		
	Total	52	513	565		

Rapid diagnostic tests for RSV and influenza were performed in 99% of the enrolled patients, and their sensitivity and specificity were 43% and 94%, and 44% and 100%, respectively

*RSV* respiratory syncytial virus, *PCR* polymerase chain reaction

### Identified viruses (Table 5)

**Fig. 2:**
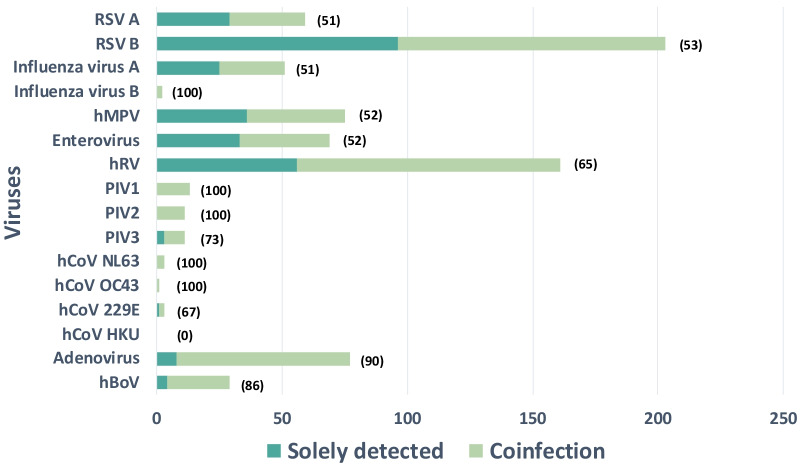
Number of viruses detected by PCR in patients with acute lower respiratory infection. The number of cases in which a specific virus was detected is shown. A single virus (green bars) and multiple viruses (light green bars) are independently shown. The numbers in parentheses refer to coinfection rates. *RSV* respiratory syncytial virus, *hMPV* human metapneumovirus, *hRV* human rhinovirus, *PIV* parainfluenza virus, *hCoV* human coronaviruses, *hBoV* human bocavirus

Viruses were detected in 88% of the patients. The number of detected and detected viruses is summarised in Table 5. RSV B (36%) and hRV (28%) were the most commonly detected viruses, followed by hMPV (13%), adenovirus (13%), enteroviruses (12%), RSV A (10%), and influenza A (9%) (Table 5 and Fig. 2). Comparing the infants (less than 12 months) and the group aged 6–12 years, RSV was more likely to be detected in infants (198 [52%] vs 54 [29%], P < 0.001). In contrast, influenza was less likely to be detected in infants (26 [7%] vs 27 [14%], P = 0.004).

**Table 5:** Viral aetiologies and rates of pneumococcal colonisation according to age and severity/mortality (n = 570)

		Total N (%)	Age category (%)			Case category (%)		
			1–11 months old	1–5 years old	6–12 years old	Non-severe cases	Severe cases	Fatal cases
	Total N (%)	570	381 (67)	178 (31)	11 (2)	463 (81)	107 (19)	28 (5)
Detected number(s) of virus(es)	No virus	68 (12)	48 (13)	18 (10)	2 (18)	57 (12)	11 (10)	3 (11)
	1 virus	291 (51)	196 (51)	88 (49)	7 (64)	233 (50)	58 (54)	20 (71)
	2 viruses	163 (29)	106 (28)	56 (31)	1 (9)	131 (28)	32 (30)	2 (7)
	3 viruses	43 (8)	27 (7)	15 (8)	1 (9)	38 (8)	5 (5)	3 (11)
	4–5 viruses	5 (1)	4 (1)	1 (1)	0 (0)	4 (1)	1 (1)	0 (0)
Detected virus*	RSV (Any)	252 (44)	198 (52)	54 (30)	0 (0)	206 (44)	46 (43)	7 (25)
	RSV A	59 (10)	48 (13)	11 (6)	0 (0)	46 (10)	13 (12)	3 (11)
	RSV B	203 (36)	158 (41)	45 (25)	0 (0)	167 (36)	36 (34)	6 (21)
	Influenza virus (Any)	53 (9)	26 (7)	24 (13)	3 (27)	41 (9)	12 (11)	8 (29)
	Influenza virus A	51 (9)	25 (7)	23 (13)	3 (27)	39 (8)	12 (11)	8 (29)
	Influenza virus B	2 (0)	1 (0)	1 (1)	0 (0)	2 (0)	0 (0)	0 (0)
	hMPV	75 (13)	40 (10)	34 (19)	1 (9)	64 (14)	11 (10)	3 (11)
	Enterovirus	69 (12)	36 (9)	32 (18)	1 (9)	57 (12)	12 (11)	4 (14)
	hRV	161 (28)	123 (32)	35 (20)	3 (27)	126 (27)	35 (33)	4 (14)
	PIV (Any)	32 (6)	18 (5)	14 (8)	0 (0)	29 (6)	3 (3)	1 (4)
	PIV1	13 (2)	8 (2)	5 (3)	0 (0)	11 (2)	2 (2)	1 (4)
	PIV2	11 (2)	5 (1)	6 (3)	0 (0)	11 (2)	0 (0)	0 (0)
	PIV3	11 (2)	7 (2)	4 (2)	0 (0)	10 (2)	1 (1)	1 (4)
	hCoV NL63	3 (1)	1 (0)	2 (1)	0 (0)	3 (1)	0 (0)	0 (0)
	hCoV OC43	1 (0)	1 (0)	0 (0)	0 (0)	1 (0)	0 (0)	0 (0)
	hCoV 229E	3 (1)	2 (1)	0 (0)	1 (9)	2 (0)	1 (1)	0 (0)
	hCoV HKU	0 (0)	0 (0)	0 (0)	0 (0)	0 (0)	0 (0)	0 (0)
	Adenovirus	76 (13)	34 (9)	39 (22)	3 (27)	65 (14)	11 (10)	2 (7)
	hBoV	29 (5)	17 (4)	12 (7)	0 (0)	21 (5)	8 (7)	1 (4)
Pneumococcal colonization		259 (45)	170 (45)	83 (47)	6 (55)	217 (47)	42 (39)	9 (32)

^*^If two or more viruses were detected in one patient, each detected virus was counted and listed in the table

All categorical data are presented as number (percentage, %)

*Total N* total number, *RSV* respiratory syncytial virus, *hMPV* human metapneumovirus, *hRV* human rhinovirus, *PIV* parainfluenza virus, *hCoV* human coronaviruses, *hBoV* human bocavirus, *PCR* polymerase chain reaction

In total, 12% (68/570) of the patients had no virus detected on RT-PCR, while 18% (12/68) were positive for RSV on the rapid test but were negative on PCR. The median age of the undetected cases was 8 months (IQR: 4–15 months), which was not significantly different from the detected cases (P = 0.91).

The epidemiological curves of representative viruses, including RSV, hRV, and influenza viruses, are shown in Fig. 1. RSV predominated in both 2017 and 2018, with a peak in September. The detection of influenza started in June 2017, and the detection rate was lower in 2018. hRV has been detected throughout the years, with peaks in August 2017 and September 2018.

### Pneumococcal colonisation

In total, 45% (259/570) of the patients were PCR-positive for pneumococcus (Table 5). Among those who had received the 10-valent PCV, 49% (130/264) were positive, which did not significantly differ from those who had not received the vaccine (42%, 129/306) (P = 0.09).

No significant differences were observed between the two groups (P = 0.15, P = 0.15, respectively) when we compared the positive rates for pneumococcus between the severe (39%) vs non-severe cases (47%) and the fatal (32%) vs non-fatal cases (46%).

### Clinical symptoms and signs of the ALRI patients detected with a single virus (Table 6)

The clinical symptoms and signs of ALRI classified by each single detected virus are summarised in Table 6. Comparing the symptoms and signs of the patients infected with each virus and those infected with a single virus, patients infected with RSV were more likely to develop rhonchi (99 [79%] vs 95 [57%], P < 0.001), and those infected with hMPV were less likely to develop prolonged capillary refill time (0 [0%] vs 23 [90%], P = 0.042). Furthermore, those infected with adenovirus had higher rates of coarse crackles (7 [88%] vs 143 [51%], P = 0.040). Other significant findings included higher rates of fever in those infected with influenza (16 [64%] vs 107 [40%], P = 0.021) and higher rates of cyanosis in those infected with hBoV (3 [75%] vs 34 [12%], P = 0.007) than in those infected with viruses other than the virus listed.

**Table 6:** Symptoms and signs of patients with ALRI due to a single virus

			Detected viruses									
			Total N (%)	RSV^a^	Influenza virus^b^	hMPV^c^	Enterovirus	hRV^d^	PIV	hCoV	Adenovirus^e^	hBoV^f^
Total N (%)			291	125	25	36	33	56	3	1	8	4
Symptoms	Cough		271 (93)	119 (95)	25 (100)	32 (89)	31 (94)	51 (91)	3 (100)	1 (100)	5 (63)	4 (100)
	Difficult breathing		215 (74)	92 (74)	16 (64)	25 (69)	26 (79)	44 (79)	2 (67)	1 (100)	5 (63)	4 (100)
	Rhinorrhea		122 (42)	60 (48)	15 (60)	13 (36)	17 (52)	13 (23)	1 (33)	0 (0)	1 (13)	2 (50)
Vital signs	Tachycardia		106 (36)	36 (29)	8 (32)	19 (53)	14 (42)	19 (34)	2 (67)	1 (100)	4 (50)	3 (75)
	Tachypnoea		223 (77)	94 (75)	17 (68)	27 (75)	29 (88)	41 (73)	3 (100)	1 (100)	7 (88)	4 (100)
	Fever ≥ 38℃		123 (42)	46 (37)	16 (64)	17 (47)	17 (52)	20 (36)	1 (33)	0 (0)	4 (50)	2 (50)
	Hypoxaemia ≤ 90%		41 (14)	17 (14)	5 (20)	3 (8)	5 (15)	6 (11)	0 (0)	1 (100)	3 (38)	1 (25)
Clinical signs	Chest indrawing		196 (67)	88 (70)	15 (60)	24 (67)	24 (73)	37 (66)	1 (33)	0 (0)	4 (50)	3 (75)
	Coarse crackles		150 (52)	61 (49)	11 (44)	24 (67)	16 (48)	26 (46)	2 (67)	0 (0)	7 (88)	3 (75)
	Wheezing		104 (36)	50 (40)	12 (48)	9 (25)	15 (45)	16 (29)	0 (0)	0 (0)	1 (13)	1 (25)
	Rhonchi		194 (67)	99 (79)	14 (56)	16 (44)	18 (55)	39 (70)	2 (67)	0 (0)	2 (25)	4 (100)
	Asymmetry of lung sounds		8 (3)	0 (0)	3 (12)	1 (3)	3 (9)	0 (0)	0 (0)	0 (0)	1 (13)	0 (0)
	Normal lung sounds		17 (6)	1 (1)	6 (24)	2 (6)	3 (9)	4 (7)	0 (0)	1 (100)	0 (0)	0 (0)
	Grunting		66 (23)	30 (24)	6 (24)	7 (19)	5 (15)	16 (29)	0 (0)	0 (0)	1 (13)	1 (25)
	Cyanosis		37 (13)	10 (8)	6 (24)	3 (8)	6 (18)	8 (14)	0 (0)	0 (0)	1 (13)	3 (75)
	Capillary refill time ≥ 3 s		23 (8)	9 (7)	3 (12)	0 (0)	3 (9)	6 (11)	0 (0)	0 (0)	1 (13)	1 (25)

Only single virus-infected patients were counted and listed in the table

All categorical data are presented as number (percentage, %)

*ALRI* acute lower respiratory infection, *Total N* total number, *RSV* respiratory syncytial virus, *hMPV* human metapneumovirus, *hRV* human rhinovirus, *PIV* parainfluenza virus, *hCoV* human coronaviruses, *hBoV* human bocavirus, *PCR* polymerase chain reaction

RSV-infected patients were less likely to develop tachycardia (36 [29%] vs 70 [42%], P = 0.019) and cyanosis (10 [8%] vs 27 [16%], P = 0.036) but were more likely to develop rhonchi (99 [79%] vs 95 [57%], P < 0.001), compared to those infected with RSV alone

The influenza virus-infected patients had higher rates of fever (16 (64%) vs 107 (40%), P = 0.021), compared to those infected with a virus other than influenza

The hMPV-infected patients had higher rates of tachycardia (19 [53%] vs 87 [34%], P = 0.029), lower rates of rhonchi (16 [44%] vs 178 [70%], P = 0.003), and prolonged capillary refill time (0 [0%] vs 23 [90%], P = 0.042), compared to those infected with hMPV

The hRV-infected patients had lower rates of rhinorrhea (13 (23%) vs 109 (46%), P = 0.002), compared to those infected with a virus other than hRV.

The adenovirus-infected patients had lower rates of cough (5 [63%] vs 266 [94%], P = 0.013) and rhonchi (2 [25%] vs 192 [68%], P = 0.018) and higher rates of coarse crackles (7 [88%] vs 143 [51%], P = 0.040), compared to those infected with adenovirus

The hBoV-infected patients had higher rates of cyanosis (3 [75%] vs 34 [12%], P = 0.007) than those infected with hBoV

### ALRI patients detected with two or more viruses (Tables 5 and 7)

Two or more viruses were detected in 211 patients (37%) (Table 5 and Fig. 2), with the most common combinations being RSV and hRV (31%), followed by hRV and adenovirus (9%), and hRV and hMPV (7%) (Table 7). Among the cases with two or more viruses detected, 2.3% of the patients were fatal. There were no dominant viruses or specific combinations of the viruses specifically identified in fatal cases (Table 7).

**Table 7:** Viral combinations in patients with acute lower respiratory infection and in-hospital mortality

Combination	Number	Length of hospitalization [day][IQR]	In-hospital mortality
RSV + hRV	51	4.0 [3.0–5.8]	0 (0)
hRV + Adenovirus	14	4.0 [3.0–6.0]	0 (0)
hMPV + hRV	12	3.5 [2.0–5.0]	1 (8)
Enterovirus + Adenovirus	10	4.0 [3.0–4.0]	0 (0)
Influenza + Adenovirus	9	5.0 [3.0–10.0]	1 (11)
RSV + Enterovirus	8	6.0 [3.8–8.3]	0 (0)
RSV + PIV	8	4.5 [3.0–5.3]	0 (0)
RSV + Adenovirus	8	5.0 [3.8–6.3]	0 (0)
hMPV + Adenovirus	6	3.5 [3.0–4.8]	0 (0)
RSV + Influenza	5	3.0 [3.0–3.8]	0 (0)
hRV + hBoV	5	5.0 [3.0–5.0]	0 (0)
RSV + hMPV	4	4.5 [2.8–8.0]	0 (0)
RSV + hBoV	4	7.5 [5.3–11.3]	0 (0)
Influenza + Enterovirus	2	4.0 [3.5–4.5]	0 (0)
hMPV + Enterovirus	2	4.0 [4.0–4.0]	0 (0)
Enterovirus + PIV	2	3.5 [2.8–4.3]	0 (0)
Adenovirus + hBoV	2	4.0 [4.0–4.0]	0 (0)
RSV + hCoV	1	1.0	0 (0)
Influenza + hMPV	1	7.0	0 (0)
Influenza + hRV	1	7.0	0 (0)
hMPV + PIV	1	3.0	0 (0)
Enterovirus + hBoV	1	14.0	0 (0)
hRV + PIV	1	4.0	0 (0)
PIV + Adenovirus	1	16.0	0 (0)
PIV + hBoV	1	14.0	0 (0)
hCoV + hBoV	1	22.0	0 (0)

Only two kinds of virus-infected patients were counted and listed in the table

Unlisted combinations, including influenza + PIV, influenza + hCoV, influenza + hBoV, hMPV + hCoV, hMPV + hBoV, enterovirus + hRV, enterovirus + hCoV, hRV + hCoV, PIV + hCoV, and adenovirus + hCoV, were not found in this study

All categorical data are presented as numbers (percentage, %). Continuous data are presented as median (interquartile range)

*Total N* total number, *RSV* respiratory syncytial virus, *hMPV* human metapneumovirus, *hRV* human rhinovirus, *PIV* parainfluenza virus, *hCoV* human coronaviruses, *hBoV* human bocavirus

Next, we evaluated the clinical impact of the number of viruses detected on the clinical outcomes. The in-hospital mortality did not differ among those detected with one virus (7%), two viruses (1%) (P = 0.07), or 3–5 viruses (6%) (P = 0.59). Similarly, the median length of hospitalisation did not differ significantly among those detected with one virus (4.0 days [IQR: 3.0–7.0 days]), two viruses (4.0 days [IQR: 3.0–6.0 days], P = 0.98), or 3–5 viruses (4.5 days [IQR: 3.0–7.0 days], P = 0.57) (Additional file 1: Table S3).

Next, we analysed the mortality and median length of hospitalisation in the patients detected with RSV only and those detected with RSV and hMPV and hBoV, which are often detected simultaneously with RSV. The mortality of those infected with RSV with or without hMPV (4% vs 0%, P = 0. 66) or hBoV (25% v. 0%, P = 0.22) did not differ significantly. Similarly, the median length of hospitalisation among the patients infected with RSV with or without hMPV (4.0 days [IQR: 3.0–5.0 days] vs 4.0 days [IQR: 3.0–6.0 days], P = 0.90) or hBoV (7.5 days [IQR: 3.0–14.2 days] vs 3.5 days [IQR: 3.0–5.8 days], P = 0.65) did not differ significantly.

### Severe ALRI patients

In total, 107 (19%) severe cases were identified. The median age did not differ between the severe (8 months [IQR: 3–12 months]) and non-severe cases (8 months, [IQR: 4–16 months]) (P = 0.39). The rate of cases that did not receive any routine immunisation was higher in the severe (22%) than in the non-severe cases (10%) (P = 0.01). A single virus was detected in 58 (54%) patients with severe cases, with RSV B (34%) and hRV (33%) being the most common. Among the severe cases, 20/107 (19%) died during hospitalisation, while there were only 8/463 (2%) mortalities in the non-severe cases (P < 0.001).

### Fatal ALRI patients

In total, 28 patients (4.9%) died. The median age did not differ between the fatal (7 months, IQR: 3–21 months) and non-fatal cases (8 months, IQR: 4–15 months) (P = 0.88). Stunting (39% [11/28] vs 23% [127/542], P = 0.056) and lack of immunisation (32% [9/28] vs 12% [63/542], P = 0.050) were more frequently observed in the fatal cases than in non-fatal cases.

Significant differences were observed in all the vital signs on admission between the fatal and non-fatal cases. Compared to the non-fatal cases, the fatal cases had a higher heart rate (160 bpm [IQR: 140–168 bpm] vs 140 bpm [IQR: 120–152 bpm], P = 0.001), body temperature (38.7 °C [IQR: 37.8–39.0 °C] vs 37.7 °C [IQR: 37.0–38.3 °C], P < 0.001), respiratory rate (69 bpm [IQR: 62–78 bpm], vs 60 bpm [IQR: 48–64 bpm], P < 0.001), and lower SpO_2_ (92% [IQR: 80–98%)] vs 96% [IQR: 94–98%], P = 0.024).

On physical examination, the following were more frequently observed in the fatal cases than in the non-fatal cases: coarse crackles (93% vs 49%, P < 0.001), wheezing (61% vs 36%, P = 0.008), grunting (75% vs 20%, P < 0.001), cyanosis (71% vs 8%, P < 0.001), and prolonged capillary refilling time ≥ 3 s (11 [39%] vs 39 [7%], P < 0.001).

In 25/28 (89%) fatal cases, at least one virus was detected: one virus (n = 20, 71%), two viruses (n = 2, 7%), and three viruses (n = 3, 11%). Influenza A (29%) and RSV B (21%) were the most commonly detected viruses.

## Discussion

The current study conducted, to the best of our knowledge, the first comprehensive investigation of hospitalised children with viral ALRI in Myanmar. The overall mortality rate in this study was 4.9%, higher than the rates reported by previous studies conducted in both developing and developed countries among hospitalised children with ALRI. The case-fatality ratios due to severe ALRI in admitted children younger than 5 years were estimated to be 2.3% (1.6–3.4%) in developing countries and 0.6% (0.4–0.8%) in developed countries [45], with an exception in eastern Mediterranean countries (7.6%; 4.9–7.9%) [45]. The factors affecting mortality were also investigated, including underlying neurological diseases [46], high rates of severe stunting (21%) [46], or insufficient data [45, 47]. In the current study, the rate of stunting was high (25%), though it did not differ significantly between fatal and non-fatal cases. Of note, stunting was the leading risk factor for ALRI mortality among children younger than 5 years, responsible for 61.4% of deaths due to ALRI [13, 17].

All vital signs were significantly different between the fatal and non-fatal cases. Additionally, abnormal respiratory sounds, grunting, cyanosis, and prolonged capillary refill time were more frequently observed in the fatal cases. As a screening tool for severe ALRI, none of the vital signs provided conclusive evidence. For example, fever was classified in the WHO guideline to have low sensitivity and specificity [48]. However, the attentive measurement of vital signs and physical examination might be helpful for clinicians to predict patients with high mortality. In addition, severe cases in the current study were more likely to turn into fatal cases, highlighting the importance of the early identification of severe cases.

Notably, 89% of the fatal cases were detected with at least one virus, with influenza A (29%) being the most commonly detected virus, followed by RSV (25%). Recent global investigations have demonstrated that RSV is the leading viral aetiology of ALRI mortality in children [5], while influenza is more frequently associated with non-fatal ALRI episodes than with fatal episodes [5]. Contrary to this finding, our study demonstrated that influenza was the leading cause of death among hospitalised children with ALRI in Myanmar.

In the current study, RSV was the most frequently detected virus, followed by hRV, hMPV, and adenovirus, consistent with the results of previous studies evaluating viral aetiologies in children with ALRI from neighbouring countries, such as Bangladesh, Thailand, and India [16, 17]. In particular, RSV had the highest burden of ALRI in infants compared to other age groups, which was consistent with the findings of previous studies [14, 49]. The peak of ALRI in the current study occurred from August to October, which coincides with the second half of the rainy season. In contrast, the influenza season occurs in the first half of the rainy season which is in July or August [28, 50].

The clinical manifestations of ALRI depend on the viral aetiology. Influenza was more likely to manifest with fever, rhinorrhoea, cyanosis, and wheezing. However, overlapping clinical presentations were frequently observed in the detected viruses, as in previous studies [18, 51]. Compared to other viruses, it has also been reported that RSV-related ALRIs frequently presented with crackles, wheezing, chest indrawing, and rhinorrhoea [52–55], but these were not observed in the current study.

In the 107 severe cases, RSV was the most frequently detected virus (43%), followed by hRV (33%), influenza (11%), and EV (11%). In most studies, RSV has been characterised to have a more severe presentation than other viruses [54, 56, 57], which is consistent with the current results. However, some studies concluded that severity was not associated with a certain virus [58–60].

Our study demonstrated that 37% of patients were infected with two or more viruses from one nasopharyngeal sample. The common combinations of the viruses were RSV and hRV, followed by RSV and adenovirus, and adenovirus and hRV. The local epidemiology of these combinations may differ depending on the location, climate, and season. For example, RSV and hBoV or influenza and hBoV were reported to be the most common combinations in children with ALRI in the United Kingdom [61, 62]. The current study did not demonstrate significant differences in the in-hospital mortality and length of hospitalisation between the patients detected with one virus and those with two or more viruses. This finding was consistent with a previous systematic review and meta-analysis, which concluded that there were no differences in the clinical severity and mortality of ALRI in children detected with single or multiple viruses [63, 64].

The most important clinical question is which combination of the viruses affects morbidity and mortality. RSV, hRV, and influenza, the common causes of childhood ALRI, are associated with high morbidity and mortality as single causative pathogens [5, 65–68]. As such, coinfection with these viruses may cause higher morbidity and mortality than that of a single virus. Different pathological mechanisms could be triggered by different viruses, which facilitate or inhibit the effects of each virus by the direct interactions of viral genes or the indirect interactions resulting from alterations in the host environment or immunological interactions [69, 70]. For example, the combination of RSV and hMPV infection has been proposed to increase disease severity compared to RSV infection alone [71, 72]. Our study demonstrated that there were no significant differences in the in-hospital mortality and the length of hospitalisation between the patients with RSV infection alone and those infected with the combination of RSV and hMPV, which was similar to previous reports [73, 74]. Of note, in contrast to these results, one study in Italy demonstrated that RSV-infected patients had a longer hospitalisation and higher hypoxia rates than the combination of RSV- and hMPV-infected patients [75]. Further studies are warranted to understand the impact of coinfection with RSV and hMPV.

Similarly, several studies have focused on evaluating the impact of hBoV and its coinfection. hBoV was mainly discovered as a coinfection in Swedish children with ALRI of unknown aetiology in 2005 [76–80]. One study reported that hBoV was responsible for severe ALRI presentation in children [81], but others reported no differences [82, 83]. As for the coinfection of hBoV with other viruses, some studies have concluded that both hBoV- and RSV-infected patients had more severe presentations [84, 85] than hBoV-infected patients, while other studies indicated that the clinical presentation of the coinfection with hBoV- and RSV did not differ from hBoV infection alone [82], which was consistent with our results. Hence, the clinical significance of coinfection is still inconclusive, and further studies are needed.

Pneumococcal infection remains the leading cause of bacterial infection in children, despite the introduction of PCV [86, 87]. In Myanmar, PCV was only recently introduced. As such, the rate of pneumococcal colonisation is unknown. We found a high colonisation rate (up to 45%) in the nasopharynx. Previous studies evaluating the pneumococcal nasopharyngeal colonisation have shown similar rates, with 54.5% and 62.5% in children aged 2–59 months in India [88] and aged 1–59 months in Thailand [89], respectively. Our results demonstrated that pneumococcal colonisation was common in children from Myanmar, providing the baseline colonisation rate in the early stages of the 10-valent PCV’s introduction. The importance of PCV in Myanmar has been confirmed, and the evaluation of pneumococcal serotypes in colonised individuals is warranted for future pneumococcal vaccine strategies in children in Myanmar, given that pneumococcal serotype replacement has become a major issue worldwide [90].

Our study revealed that RSV was the most frequently detected virus, and influenza was the leading cause of death among hospitalised children with ALRI in Myanmar. Medical professionals should reaffirm the significance of these two viruses. First, the introduction of palivizumab prophylaxis is needed to reduce both the RSV-related morbidity and mortality in children in Myanmar as this can prevent serious ALRI in high-risk infants and children (e.g. premature infants ≤ 35 weeks gestational age with chronic lung disease of prematurity and congenital heart disease) [91]. Similarly, influenza vaccines can prevent hospitalisation due to influenza in children [92, 93]. Thus, the strengthening of prevention programs will improve the health of children in Myanmar, though further studies are needed to clarify the effectiveness of these prevention interventions in Myanmar. Of note, rapid diagnostic tests for RSV and influenza demonstrated low sensitivity (43% and 44%, respectively), even though these kits used in the current study was reported to have a high sensitivity, specificity, and accuracy of above 85% in other countries [94, 95]. Viral load in the nasopharyngeal specimens of the patients would be sufficient to be detected by the rapid test because a majority of the patients had a test at or 3 days after symptom onset. Therefore, evaluation and improvement of the procedure are also needed to improve the sensitivity of the tests.

The judicious use of antibiotics is urgently required in Myanmar due to the issue of antimicrobial resistance. Myanmar has been reported to have a high risk of emergence and development of antibiotic resistance due to the availability of antibiotics as over-the-counter drugs and the inappropriate use by physicians [96]. In our study, more than half of the enrolled children were prescribed antibiotics despite the suspicion of viral ALRI. Among them, one-third of the children received more than two antibiotics. Influenza, which was associated with high mortality in the current study, can present in conjunction with, or be followed by a secondary bacterial infection, more commonly *Staphylococcus aureus* [97, 98] and *Streptococcus pneumoniae* [99]. Moreover, community-acquired methicillin-resistant *Staphylococcus aureus* infections have caused high levels of morbidity and mortality in children with influenza [100]. Therefore, it is necessary to reserve antibiotics for secondary bacterial infections.

As for the general characteristics of ALRI patients who were registered in this study, the prevalence of moderate to severe stunting was 25%. Meanwhile, in the United Nations Children’s Fund (UNICEF) report, 20% of children living in urban areas in Myanmar had an equal level of stunting [26]. Moreover, the vaccination coverage rates were 66% for Hib and 46% for PCV, which are lower than the WHO-UNICEF estimates of 89% for the third dose of both Hib and PCV [24]. However, these estimates might not reflect the actual situation in Myanmar. Indeed, the Myanmar Demographic and Health Survey by the United States Agency for International Development revealed that only 55% of children aged < 2 years had complete vaccination coverage (one dose each for Bacillus Calmette-Guérin and measles and three doses each for diphtheria-pertussis-tetanus and polio), which were noticeably lower than the WHO-UNICEF estimates of routine administrative coverage of 86% for Bacillus Calmette-Guérin, 75% for diphtheria-pertussis-tetanus, 76% for oral polio vaccine, and 86% for measles-containing-vaccine first-dose [101]. In addition to nutrition and immunisation, socioeconomic factors, including maternal education, are also associated with a high risk of ALRI [13]. Thus, the awareness of parents or guardians about ALRI is essential [102]. Further economic and social developments are expected in Myanmar.

The current major concern is the Myanmar coup that began on 1 February 2021, where the military seized control and declared a year-long state of emergency. Several mass protests have been taking place in Myanmar, and many causalities have been reported. Access to medical services could be restricted due to the focused care for the injured or the limited working time for the medical professionals. We assume that the effects of this critical situation will impact child health, including care for children with ALRI.

A few limitations of this study need to be acknowledged. First, we obtained samples from the nasopharynx and not directly from the lower respiratory tract because of the difficulty in forcing children to cough to collect a sputum sample. Therefore, the results may not reflect the virus directly causing ALRI. It is important to note that the aetiologies of ALRI could also evolve from upper to lower respiratory tract infections and develop into a severe illness [103]. Second, the samples were obtained from hospitalised patients each month according to paediatricians’ decisions. Therefore, selection bias among the paediatricians may have occurred. This could also have negatively impacted the incidence of each viral infection in this population; for example, the incidence of influenza was low compared to that in studies in other countries [16, 51, 61, 104]. Lastly, the role of pneumococcal infection in ALRI could not be elucidated due to the negative bacterial culture results. The high positive rate of PCR in the current study reflects pneumococcal colonisation that does not cause disease. Further investigation of pneumococcal infection, including incidence and serotype evaluation, is necessary, given that PCV10 has been recently introduced in Myanmar.

## Conclusions

This prospective study revealed the significant impact of viral ALRI on infants and children in Myanmar owing to the high mortality. RSV B and hRV were the most commonly detected viruses from nasopharyngeal swabs, while the influenza virus and RSV B were the most frequently associated with fatal cases. The results of this study will contribute to the knowledge on the epidemiology, diagnosis, and appropriate management of ALRI in Myanmar, and it has the potential to improve morbidity and mortality of children with ALRI in the future.

## Supplementary Information

**Additional file 1: Table S1.** Sequences of primers and probes, concentration, and size of PCR products of each PCR assay. **Table S2.** Clinical characteristics based on the detected number of viruses (n = 570). **Table S3.** Clinical signs, course, and outcomes based on the detected numbers of viruses (n = 570). **Table S4.** Laboratory and radiologic findings based on the number of viruses detected (n = 570).

## Data Availability

All data generated or analysed during this study are included in this published article and its supplementary information files.

## Abbreviations

ALRI: Acute lower respiratory infection
hBoV: Human bocavirus
hRV: Human rhinovirus
PIV: Parainfluenza virus
RSV: Respiratory syncytial virus
