# Supplementary material for: Clinical manifestations and outcome of viral acute lower respiratory infection in hospitalised children in Myanmar
Source: BMC Infect Dis. 2022 Apr 8;22:350. doi: 10.1186/s12879-022-07342-1 (PMC8992414; doi:10.1186/s12879-022-07342-1)
Supplement: Supplementary file 1 — Additional file 1: Table S1. Sequences of primers and probes, concentration, and size of PCR products of each PCR assay. Table S2. Clinical characteristics based on the detected number of viruses (n = 570). Table S3. Clinical signs, course, and outcomes based on the detected numbers of viruses (n = 570). Table S4. Laboratory and radiologic findings based on the number of viruses detected (n = 570). [file 12879_2022_7342_MOESM1_ESM.docx]

**Supplementary Table 1. Sequences of primers and probes, concentration, and size of PCR products of each PCR assay**

| **Virus** | **Primers and probes** | **Sequences（5' to 3'）** | **Final concentration (μM）** | **Size of PCR product（bp)** | **Reference number** |
| --- | --- | --- | --- | --- | --- |
| **Respiratory syncytial virus A** | Forward primer | **GCTCTTAGCAAAGTCAAGTTGAATGA** | 0.5 | 82 | 32 |
|  | Reverse primer | **TGCTCCGTTGGATGGTGTATT** | 0.5 |  |  |
|  | Probe | **（FAM）ACACTCAACAAAGATCAACTTCTGTCATCCAGC（BHQ1）** | 0.2 |  |  |
| **Respiratory syncytial virus B** | Forward primer | **GATGGCTCTTAGCAAAGTCAAGTTAA** | 0.5 | 104 | 32 |
|  | Reverse primer | **TGTCAATATTATCTCCTGTACTACGTTGAA** | 0.5 |  |  |
|  | Probe | **（FAM）TGATACATTAAATAAGGATCAGCTGCTGTCATCCA（BHQ1）** | 0.2 |  |  |
| **Influenza virus A** | Forward primer | **GACCRATCYTGTCACCTCTGAC** | 0.6 | 106 | 34, 35 |
|  | Reverse primer | **AGGGCATTYTGGACAAAKCGTCTA** | 0.6 |  |  |
|  | Probe | **（FAM）TGCAGTCCTCGCTCACTGGGCACG（BHQ1）** | 0.6 |  |  |
| **Influenza virus B** | Forward primer | **AAATACGGTGGATTAAATAAAAGCAA** | 0.6 | 170 | 34, 35 |
|  | Reverse primer | **CCAGCAATAGCTCCGAAGAAA** | 0.6 |  |  |
|  | Probe | **（FAM）CACCCATATTGGGCAATTTCCTATGGC（BHQ1）** | 0.2 |  |  |
| **Human metapneumovirus** | Forward primer | **CATAYAARCATGCTATATTAAAAGAGTCTC** | 1.0 | 163 | 37 |
|  | Reverse primer | **CCTATYTCWGCAGCATATTTGTAATCAG** | 1.0 |  |  |
|  | Probe | **（FAM）CAACHGCAGTRACACCYTCATCATTRCA（BHQ1）** | 0.3 |  |  |
| **Enterovirus** | Forward primer | **GCCCCTGAATGCGGC** | 0.5 | 148 | 36, 40 |
|  | Reverse primer | **AATTGTCACCATAAGCAGC** | 0.4 |  |  |
|  | Probe | **（FAM）CGGAACCGACTACTTTGGGTGTCCGT（BHQ1）** | 0.1 |  |  |
| **Enterovirus/ Human rhinovirus** | Forward primer | **AGCCTGCGTGGCKGCC** | 0.5 | 203 | 40 |
|  | Reverse primer | **GAAACACGGACACCCAAAGTAGT** | 0.5 |  |  |
|  | Probe | **（FAM）CTCCGGCCCCTGAATGYGGCTAA（BHQ1）** | 0.1 |  |  |
| **Parainfluenza viruses 1** | Forward primer | **GTTGTCAATGTCTTAATTCGTATCAATAATT** | 0.9 | 101 | 38, 39 |
|  | Reverse primer | **GTAGCCTMCCTTCGGCACCTAA** | 0.9 |  |  |
|  | Probe | **（FAM）TAGGCCAAAGATTGTTGTCGAGACTATTCCAA（BHQ1）** | 0.2 |  |  |
| **Parainfluenza viruses 2** | Forward primer | **AGGACTATGAAAACCATTTACCTAAGTGA** | 0.6 | 157 | 38, 39 |
|  | Reverse primer | **AAGCAAGTCTCAGTTCAGCTAGATCA** | 0.6 |  |  |
|  | Probe | **（FAM）ATCAATCGCAAAAGCTGTTCAGTCACTGCTATAC（BHQ1）** | 0.1 |  |  |
| **Parainfluenza viruses 3** | Forward primer | **TGATGAAAGATCAGATTATGCATATC** | 0.6 | 249 | 38, 39 |
|  | Reverse primer | **CCGGGACACCCAGTTGTG** | 0.6 |  |  |
|  | Probe | **（FAM）TGGACCAGGGATATACTACAAAGGCAAAATAATATTTCTC（BHQ1）** | 0.1 |  |  |
| **Human coronaviruses (NL63)** | Forward primer | **AACCTCGTTGGAAGCGTGTT** | 0.3 | 61 | 40 |
|  | Reverse primer | **CGAGGACCAAAGCACTGAATAA** | 0.3 |  |  |
|  | Probe | **（FAM）ATTTTCCTCTCTGGTAG（BHQ1）** | 0.2 |  |  |
| **Human coronaviruses (OC43)** | Forward primer | **GACATGGCTGATCAAATTGCTAGT** | 0.3 | 67 | 40 |
|  | Reverse primer | **GCTGAGGTTTAGTGGCATCCTT** | 0.3 |  |  |
|  | Probe | **（FAM）TCTGGCAAAACTTGG（BHQ1）** | 0.2 |  |  |
| **Human coronaviruses (229E)** | Forward primer | **CTGCCAAGAGTCTTGCTCGTT** | 0.3 | 80 | 40 |
|  | Reverse primer | **TCTTTTCCACCGTGGCTTTT** | 0.3 |  |  |
|  | Probe | **（FAM）AGAACAAAAGCATGAAATG（BHQ1）** | 0.2 |  |  |
| **Human coronaviruses (HKU)** | Forward primer | **CCCGCAAACATGAATTTTGTT** | 0.3 | 61 | 40 |
|  | Reverse primer | **CATTCATTCGCAAGGCGATA** | 0.3 |  |  |
|  | Probe | **（FAM）AATCTATCACCATGTGAA（BHQ1）** | 0.2 |  |  |
| **Adenovirus 2*** | Forward primer | **CCAGGACGCCTCGGAGTA** | 0.4 | 85 | 41 |
|  | Reverse primer | **AAACTTGTTATTCAGGCTGAAGTACGT** | 0.4 |  |  |
|  | Probe | **（FAM）AGTTTGCCCGCGCCACCG（BHQ1）** | 0.2 |  |  |
| **Adenovirus 4**** | Forward primer | **GGACAGGACGCTTCGGAGTA** | 0.4 | 84 | 41 |
|  | Reverse primer | **CTTGTTCCCCAGACTGAAGTAGGT** | 0.4 |  |  |
|  | Probe | **（FAM）CAGTTCGCCCGYGCMACAG（BHQ1）** | 0.2 |  |  |
| **Human bocavirus** | Forward primer | **AGCATCGCTCCTACAAAAGAAAAG** | 0.3 | 201 | 33 |
|  | Reverse primer | **TCTTCATCACTTGGTCTGAGGTCT** | 0.3 |  |  |
|  | Probe | **（FAM）AGGCTCGGGCTCATATCATCAGGAACA（BHQ1）** | 0.1 |  |  |

*Adenovirus 2 set detects Adenovirus 1, 2, 5, 6, 7, 8, 10, 19, 40, and 41.

** Adenovirus 4 set detects Adenovirus 1, 3, 4, 5, 6, 7, and 40.

**Supplementary Table 2. Clinical characteristics based on the detected number of viruses (n = 570)**

|  | | **Total (%)** | **Detected number(s) of virus(es) (%)** | | | | |
| --- | --- | --- | --- | --- | --- | --- | --- |
|  |  |  | **No virus** | **1 virus** | **2 viruses** | **3 viruses** | **4-5 viruses** |
|  |  | **570** | **68 (12)** | **291 (51)** | **163 (29)** | **43 (8)** | **5 (1)** |
| **Characteristics** | Median Age [months] [IQR] | **8.0 [4.0-15.0]** | 8.0 [4.0-16.0] | 8.0 [4.0-14.0] | 8.0 [4.0-15.0] | 10.0 [3.0-18.0] | 7.0 [3.0-13.0] |
|  | Male | **322 (56)** | 38 (56) | 156 (54) | 101 (62) | 24 (56) | 3 (60) |
|  | Weight-for-age Z score |  |  |  |  |  |  |
|  | Severe (< -3) | **66 (12)** | 14 (21) | 31 (11) | 15 ( 9 ) | 5 (12) | 1 (20) |
|  | Moderate ( ≥ -3 to < -2) | **72 (13)** | 5 ( 7 ) | 34 (12) | 26 (16) | 6 (14) | 1 (20) |
|  | Normal (≥ -2) | **432 (76)** | 49 (72) | 226 (78) | 122 (75) | 32 (74) | 3 (60) |
|  | Underling Diseases | **73 (13)** | 15 (22) | 41 (14) | 15 ( 9 ) | 2 ( 5 ) | 0 ( 0 ) |
|  | Smoker in Family | **243 (43)** | 27 (40) | 128 (44) | 67 (41) | 20 (47) | 1 (20) |
| **Immunization** | DPT | **422 (74)** | 50 (74) | 228 (78) | 109 (67) | 31 (72) | 4 (80) |
|  | Hib | **374 (66)** | 47 (69) | 205 (70) | 91 (56) | 29 (67) | 2 (40) |
|  | BCG | **460 (81)** | 53 (78) | 241 (83) | 125 (77) | 37 (86) | 4 (80) |
|  | MR | **138 (24)** | 16 (24) | 73 (25) | 33 (20) | 16 (37) | 0 ( 0 ) |
|  | PCV | **264 (46)** | 28 (41) | 144 (49) | 68 (42) | 23 (53) | 1 (20) |
|  | None | **72 (13)** | 11 (16) | 32 (11) | 23 (14) | 6 (14) | 0 ( 0 ) |
| **History** | Sick contact | **65 (11)** | 4 ( 6 ) | 38 (13) | 20 (12) | 3 ( 7 ) | 0 ( 0 ) |
|  | Symptoms Onset [Days] [IQR] | **3.0 [1.5-4.0]** | 3.0 [2.0-4.0] | 3.0 [2.0-4.0] | 2.5 [1.0-3.3] | 2.0 [1.0-4.0] | 3.0 [2.0-3.0] |
| **Symptoms** | Cough | **534 (94)** | 64 (94) | 271 (93) | 156 (96) | 38 (88) | 5 (100) |
|  | Difficult Breathing | **415 (73)** | 43 (63) | 215 (74) | 123 (75) | 32 (74) | 2 (40) |
|  | Rhinorrhea | **247 (43)** | 28 (41) | 122 (42) | 73 (45) | 23 (53) | 1 (20) |

All categorical data are presented as numbers (percentage, %). Continuous data are presented as median (interquartile range).

Abbreviations: ALRI, acute lower respiratory infection; Total N, total number; IQR, interquartile range; DPT, a combination vaccine of diphtheria, pertussis, and tetanus; Hib: *Haemophilus influenzae* type b vaccine; BCG, Bacille Calmette-Guérin vaccine; MR, Measles-rubella vaccine; PCV, pneumococcal conjugate vaccines.

**Supplementary Table 3. Clinical signs, course, and outcomes based on the detected numbers of viruses (n = 570)**

|  |  | **Total N (%)** | **Detected number(s) of virus(es)* (%)** | | | | |
| --- | --- | --- | --- | --- | --- | --- | --- |
|  |  |  | **No virus** | **1 virus** | **2 viruses** | **3 viruses** | **4-5 viruses** |
|  |  |  |  |  |  |  |  |
| **Total N (%)** | | **570** | **68 (12)** | **291 (51)** | **163 (29)** | **43 (8)** | **5 (1)** |
| **Vital Signs** | Tachycardia | **208 (36)** | 24 (35) | 106 (36) | 61 (37) | 14 (33) | 3 (60) |
|  | Heart Rate [/m] [IQR] | **140 [122-152]** | 140 [128-150] | 140 [123-156] | 140 [126-151] | 128 [118-144] | 170 [140-195] |
|  | Tachypnoea | **443 (78)** | 54 (79) | 223 (77) | 127 (78) | 35 (81) | 4 (80) |
|  | Respiratory Rate [/m] [IQR] | **60 [48-64]** | 60 [50-62] | 60 [48-64] | 60 [48-66] | 60 [48-68] | 52 [48-55] |
|  | Fever ≥ 38℃ | **232 (41)** | 28 (41) | 123 (42) | 63 (39) | 16 (37) | 2 (40) |
|  | Body Temperature [℃] [IQR] | **37.8 [37.2-38.3]** | 37.8 [37.2-38.3] | 37.9 [37.2-38.5] | 37.8 [37.2-38.3] | 37.8 [37.2-38.3] | 38.0 [37.5-38.6] |
|  | Hypoxaemia ≤ 90% | **70 (12)** | 7 (10) | 41 (14) | 21 (13) | 1 ( 2 ) | 0 ( 0 ) |
|  | Oxgen Saturation [%] [IQR] | **96 [94-98]** | 97 [94-98] | 96 [94-98] | 96 [94-98] | 97 [96-98] | 94 [92-97] |
| **Clinical Signs** | Chest indrawing | **383 (67)** | 44 (65) | 196 (67) | 108 (66) | 32 (74) | 3 (60) |
|  | Coarse crackles | **291 (51)** | 38 (56) | 150 (52) | 81 (50) | 19 (44) | 3 (60) |
|  | Wheezing | **212 (37)** | 16 (24) | 104 (36) | 73 (45) | 17 (40) | 2 (40) |
|  | Rhonchi | **357 (63)** | 36 (53) | 194 (67) | 96 (59) | 30 (70) | 1 (20) |
|  | Asymmetry of lung sounds | **18 ( 3 )** | 5 ( 7 ) | 8 ( 3 ) | 3 ( 2 ) | 2 ( 5 ) | 0 ( 0 ) |
|  | Normal lung sounds | **29 ( 5 )** | 3 ( 4 ) | 17 ( 6 ) | 7 ( 4 ) | 2 ( 5 ) | 0 ( 0 ) |
|  | Grunting | **130 (23)** | 13 (19) | 66 (23) | 38 (23) | 12 (28) | 1 (20) |
|  | Cyanosis | **65 (11)** | 6 ( 9 ) | 37 (13) | 16 (10) | 5 (12) | 1 (20) |
|  | Capillary refill time ≥ 3 sec | **50 ( 9 )** | 6 ( 9 ) | 23 ( 8 ) | 14 ( 9 ) | 7 (16) | 0 ( 0 ) |
| **Treatmnet** | Antibiotics | **325 (57)** | 43 (63) | 164 (56) | 90 (55) | 25 (58) | 3 (60) |
|  | 2 or more than 2 drugs | **107 (19)** | 18 (26) | 53 (18) | 25 (15) | 10 (23) | 1 (20) |
|  | Anti-viral Treatment | **12 ( 2 )** | 1 ( 1 ) | 6 ( 2 ) | 3 ( 2 ) | 2 ( 5 ) | 0 ( 0 ) |
|  | ICU Management | **51 ( 9 )** | 5 ( 7 ) | 27 ( 9 ) | 12 ( 7 ) | 6 (14) | 1 (20) |
|  | O_2_ Supply | **328 (58)** | 40 (59) | 169 (58) | 101 (62) | 16 (37) | 2 (40) |
|  | Mechanical Ventilation | **39 ( 7 )** | 4 ( 6 ) | 22 ( 8 ) | 7 ( 4 ) | 5 (12) | 1 (20) |
|  | CPAP | **14 ( 2 )** | 3 ( 4 ) | 8 ( 3 ) | 3 ( 2 ) | 0 ( 0 ) | 0 ( 0 ) |
|  | Fluid infusion | **176 (31)** | 24 (35) | 84 (29) | 54 (33) | 12 (28) | 2 (40) |
| **Outcomes** | In-hospital mortality | **28 ( 5 )** | 3 ( 4 ) | 20 ( 7 ) | 2 ( 1 ) | 3 ( 7 ) | 0 ( 0 ) |
|  | Sequelae | **8 ( 1 )** | 3 ( 4 ) | 3 ( 1 ) | 2 ( 1 ) | 0 ( 0 ) | 0 ( 0 ) |
|  | Length of hospitalization [day][IQR] | **4.0 [3.0-7.0]** | 5.0 [3.0-7.5] | 4.0 [3.0-7.0] | 4.0 [3.0-6.0] | 5.0 [3.0-7.0] | 4.0 [3.0-5.0] |

All categorical data are presented as numbers (percentage, %). Continuous data are presented as median (interquartile range).

Abbreviations: Total N, total number; IQR, interquartile range; ICU, intensive care unit; CPAP, continuous positive airway pressure

**Supplementary Table 4. Laboratory and radiologic findings based on the number of viruses** **detected (n = 570)**

|  |  | **Total N (%)** | **Detected number(s) of virus(es) (%)** | | | | |
| --- | --- | --- | --- | --- | --- | --- | --- |
|  |  |  | **No virus** | **1 virus** | **2 viruses** | **3 viruses** | **4-5 viruses** |
|  |  |  |  |  |  |  |  |
| **Total N (%)** | | **570** | **68 (12)** | **291 (51)** | **163 (29)** | **43 (8)** | **5 (1)** |
| **CBC** | **Numbers of tests performed (%)** | **361 (63)** | 48 (71) | 174 (60) | 108 (66) | 26 (60) | 5 (100) |
|  | WBC [x10^3^/μL] [IQR] | **13.4 [9.7-18.6]** | 14.4 [9.3-19.2] | 13.6 [9.6-18.6] | 12.6 [9.6-17.1] | 15.1 [10.6-20.4] | 15.1 [10.6-18.2] |
|  | Neutrophil [%] | **50 [37-69]** | 51 [37-70] | 51 [37-69] | 50 [37-69] | 51 [38-70] | 56 [42-70] |
|  | Hemoglobin [g/dL] [IQR] | **10.4 [9.6-11.2]** | 10.7 [9.8-11.4] | 10.3 [9.5-11.0] | 10.3 [9.6-11.3] | 10.8 [10.2-12.1] | 10.7 [9.9-11.2] |
|  | Platelets [x10^3^/µL] [IQR] | **393 [285-507]** | 400 [276-488] | 386 [285-498] | 414 [301-522] | 410 [354-500] | 443 [319-553] |
| **Chemistry** | **Numbers of tests performed (%)** | **231 (41)** | 33 (49) | 112 (38) | 69 (42) | 14 (33) | 3 (60) |
|  | C-reactive protein [mg/dL][IQR] | **1.6 [0.3-4.6]** | 1.5 [0.4-3.5] | 1.6 [0.4-4.5] | 1.5 [0.1-4.1] | 5.8 [0.3-8.1] | 0.1 [0.1-1.1] |
| **Chest X-ray** | **Numbers of tests performed (%)** | **336 (59)** | 48 (71) | 158 (54) | 95 (58) | 31 (72) | 4 (80) |
|  | Consolidation | **148 (44)** | 20 (42) | 71 (45) | 42 (44) | 13 (42) | 2 (50) |
|  | Overinflation | **144 (43)** | 14 (29) | 71 (45) | 43 (45) | 14 (45) | 2 (50) |
|  | Normal Finding | **36 (11)** | 8 (17) | 17 (11) | 7 ( 7 ) | 4 (13) | 0 ( 0 ) |

All categorical data are presented as numbers (percentage, %). Continuous data are presented as median (interquartile range).

Abbreviations: Total N, total number; IQR, interquartile range; CBC, complete blood count; WBC, white blood cells; RSV, respiratory syncytial virus; PCR, polymerase chain reaction.
